# Complex Interplay Between COVID-19 Lockdown and Myopic Progression

**DOI:** 10.3389/fmed.2022.853293

**Published:** 2022-03-21

**Authors:** Tao Cai, Lianghui Zhao, Ling Kong, Xianli Du

**Affiliations:** ^1^Shandong First Medical University and Shandong Academy of Medical Sciences, Jinan, China; ^2^State Key Laboratory Cultivation Base, Shandong Provincial Key Laboratory of Ophthalmology, Eye Institute of Shandong First Medical University, Qingdao, China; ^3^Qingdao Eye Hospital of Shandong First Medical University, Qingdao, China

**Keywords:** myopia, COVID-19, e-learning, asthenopia, home confinement

## Abstract

**Purpose:**

To compare the myopic progression before and during strict home confinement when coronavirus disease 2019 (COVID-19) outbreak and explore the potential influencing factors.

**Methods:**

A cross-sectional study. One hundred and fifteen myopic children (115 right eyes) who replace their frame-glasses from December 2019 to January 2020 and with complete refractive records in our hospital since myopia were involved in the study. At the beginning of the strict home confinement and after a 3-month strict home confinement during the COVID-19 pandemic, they were invited to our hospital to examine the axial length and refractive errors. And visual functions, convergence insufficiency symptom survey (CISS) scale and questionnaires were also performed. Besides, the axial length and refractive errors before the COVID-19 were got from outpatient case files. The effect of strict home confinement on myopia was assessed by comparing monthly axial elongation before COVID-19 and during strict home confinement. Spearman correlation analysis was performed to explore the correlation between potential influencing factors and myopia progression.

**Results:**

Axial length's monthly elongation during strict home confinement was 35% higher than normal periods (0.046 vs. 0.033 mm/month, *P* = 0.003). The proportion of severe asthenopia doubled (*P* = 0.020). For myopia progression, heredity, close indoor work time and electronic products were risk factors. Besides, the protective factors were age, rest time after continuous eye usage, sleep time and distance from eye to computer screen.

**Conclusions:**

During COVID-19, the decline in outdoor activities and increase of exposure time to digital screens accelerated the progression of myopia by 1/3.

## Introduction

Myopia has become a serious global problem. Studies had shown that 49.8% of the global population will become myopic, and 9.8% will become high myopic by 2050 (1). Besides, by 2050, 84% of the Chinese children and children will suffer from myopia (2). Many factors are related to the occurrence and development of myopia, and the prevention and control of myopia has attracted great attention. In December 2019, the coronavirus disease (COVID-19) spread rapidly in China and around the world. To prevent the spread and outbreak of COVID-19, the Chinese government closed schools and implemented home confinement measures across the country from early February to mid-May 2020. Affected by the home confinement measures, the modes of learning changed from traditional offline/in-class studies in schools to online learning through digital platforms. Additionally, a decline in outdoor activities and increased exposure time to digital screens have raised concerns about “quarantine myopia” (3, 4).

Myopia is a complex multifactorial disorder, which influenced by risk factors such as longtime of close work and less light exposure (5). During special situations of home confinement, factors such as increased exposure time to electronic devices (online learning through digital platforms and daily homework), changed patterns of activities, imbalanced diet and sleep duration can affect the progress of myopia (6). Studying the complex relationship between home confinement during the COVID-19 pandemic and the development of myopia have a unique significance in helping prevent and control the progress of myopia. However, most studies about the COVID-19 and myopia were epidemiological studies with a large time span (usually using the academic year as the node). And the results were mostly the incidence rate or elongation of myopia, which cannot accurately reflect the progress of myopia caused by the strict home confinement measures during the COVID-19 pandemic. During the COVID-19 outbreak in China, the strict home confinement measures lasted approximately 3 months (early February to mid-May 2020). At present, there are no study on axial length elongation and risk factors during this special confinement period. Fortunately, during the critical, peak-COVID-19 outbreak period when schools were generally closed, we recruited 115 myopic children from our hospitals to study the impact of the strict home confinement on the development of myopia. Furthermore, this study also explored the relationship between myopia progression and potential influencing factors such as eye care habits and visual function during the COVID-19 pandemic, hoping to provide guidance for prevention and control of myopia among children in the “post-COVID-19 period.”

## Methods

This study adhered to the tenets of the Declaration of Helsinki and was approved by the Ethics Committee of Shandong Eye Institute. All patients or their guardians signed informed consent forms. The data were collected from Qingdao Eye Hospital. In total, 115 myopic children (115 right eyes) who replace their frame-glasses at our hospital from December 2019 to January 2020 were successfully recruited into the study. These children had been treated in our hospital since myopia and had complete refractive records. In addition, they had not received other myopia control treatment. At the beginning of the strict home confinement (~2 weeks from early to mid-February 2020) and after a 3-month strict home confinement during the COVID-19 pandemic (~2 weeks from mid-May to the end of May 2020), they were invited to have examinations in our hospital. All patients were examined the axial length (IOL Master 700, ZEISS, Germany) and refractive errors (without cycloplegia). Besides, we got their axial length and refractive errors before the COVID-19 lockdown (mid-September 2019 to the end of January 2020, which match the time range of “strict home confinement”) from outpatient case files (Table 1).

**Table 1:** Baseline characteristics of different periods.

	**Stage 1**	**Stage 2**
Gender, male, *N* (%)	66 (57.40)	66 (57.40)
Gender, female, *N* (%)	49 (42.6)	49 (42.6)
Age	9.34 ± 2.00	9.60 ± 2.30
Spherical refractive errors (D)	−2.31 ± 1.33	−2.51 ± 1.54
Astigmatism (D)	−0.56 ± 0.94	−0.59 ± 0.98
AL (mm)	24.55 ± 1.04	24.65 ± 1.03

*Stage 1, Baseline before the COVID-19 pandemic; Stage 2, Baseline at the beginning of home confinement. AL, Axial Length. Data were expressed as mean ± standard deviations*.

All the children had no history of eye surgery, trauma, other eye diseases, or orthokeratology. And all the best-corrected visual acuity was ≥20/25. The monthly axial growth rate was used as an observation index to assess the development of myopia before the COVID-19 pandemic and after the strict home confinement during the COVID-19 pandemic. In order to explore the influencing factors about myopia progression during strict home confinement, visual functions and convergence insufficiency symptom survey (CISS) (7) as well as a questionnaire concerning eye care habits during the COVID-19 lockdown were performed on all patients at the beginning of the strict home confinement and after a 3-month strict home confinement. The subject's response to the questions of CISS were recorded. Survey scores as follows: never (0), infrequently (1), sometimes (2), fairly often (3), and always (4). The total score was summed the points of all items, which could range from 0 to 60. According to the total score, <8 was defined as no asthenopia, 9-14 as mild asthenopia, 15-28 as moderate asthenopia, and >29 as severe asthenopia.

Visual functions examinations, including positive relative accommodation (PRA), negative relative accommodation (NRA), amplitude (AMP) and accommodative response (TOPCON CV-5000, Japan), were performed. The questionnaire mainly included information about regional differences, hereditary factors, outdoor light exposure time, digital devices exposure time and distances, types of digital devices, and rest time (specific information shown in Supplementary Table 1). The questionnaires were completed by the older students themselves; in the care of the younger students, their parents assisted them.

### Statistical Analysis

Data were analyzed by Statistical Package for Social Sciences software (version 23.0). Descriptive statistics were presented as mean ± standard deviation. All the data were first examined with the Kolmogorov–Smirnov test to evaluate the normality. The paired sample *t*-test or Wilcoxon signed-rank test was used to analyze the differences according to the normal or non-normal distribution, the myopia progression rate (eye axial length) was analyzed by Mann-Whitney *U*-test. Spearman correlation analysis was performed to explore the correlation between potential influencing factors and growth rate of eye axial length. The significance level for all of the tests was set at 0.05.

## Results

### Comparison of Myopia Progression

**Figure 1:**
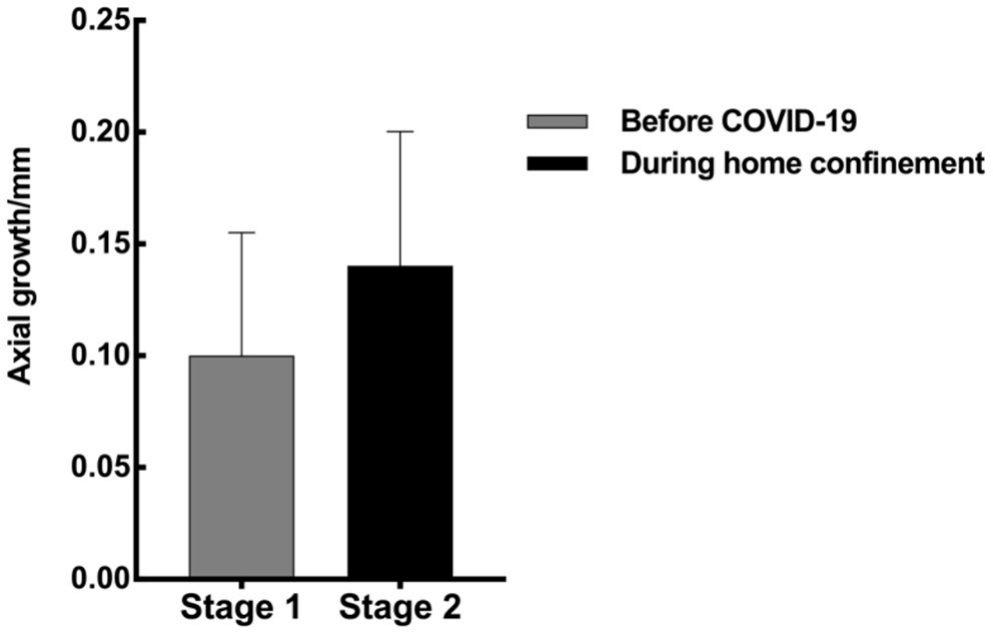
Eye axial growth before and after the COVID-19 pandemic (in 3 months, respectively).

The results showed that the monthly growth rate of eye axial length during the strict home confinement after the COVID-19 outbreak was 35% faster than that before COVID-19 (mid-September 2019 to the end of January 2020) (0.046 vs. 0.033 mm/month, *P* = 0.003; Figure 1). And refractive error (non-cycloplegic autorefraction) also increased rapidly (0.45 D vs. 0.20 D in 3 months).

### Influencing Factors of Axial Elongation After the Strict Home Confinement Measures

The monthly axial growth rate was negatively correlated with age (*r* = −0.442, *P* < 0.001), distance from eyes to computer screen (*r* = −0.238, *P* = 0.013), rest time after continuous eye-use (*r* = −0.254, *P* = 0.007), outdoor light exposure (*r* = −0.242, *P* = 0.045), and sleep duration (*r* = −0.197, *P* = 0.040); it was positively correlated with parental history of myopia (*r* = 0.305, *P* = 0.001), myopia degree of parents (*r* = 0.236, *P* = 0.011), indoor reading and writing time (*r* = 0.266, *P* = 0.004), using time of smartphones and iPads (*r* = 0.227, *P* = 0.021), time of close work (*r* = 0.370, *P* < 0.001) and methods of light exposure (*r* = 0.235, *P* = 0.014). Among these, the risk factors of myopia progression include heredity and indoor close work and the use of electronic products. The protective factors of myopia progression include age, the rest time after continuous eye-use, light exposure, more sleep time and far distance from the screen when using a computer. Other factors did not correlate with the axial growth rate (Table 2). Moreover, the method of light exposure significantly affects axial growth rate; the axial growth rate of those who choose indoor light exposure (through balcony overlooking) was 48.8% faster than those who choose outdoor light exposure (0.061 ± 0.050 vs. 0.041 ± 0.027 mm/month, *P* = 0.015).

**Table 2:** Correlation analysis of eye care habits on the growth rate of eye axial length.

	**Axial progression rate (mm/month)**	
	***r***	***P*-value**
Age	−0.442^**^	<0.001
Gender	0.127	0.178
Living region	−0.170	0.070
Whether parents are myopic	0.305^**^	0.001
The myopia degree of parent (choose the higher)	0.236^*^	0.011
Indoor reading and writing (hours/day)	0.266^**^	0.004
Reading distance	−0.109	0.245
Time of using computer(hours/day)	0.191^*^	0.041
Distance between eyes and computer screen	−0.238^*^	0.013
Time of using smartphones and iPads(hours/day)	0.227^*^	0.021
The time of close work (including reading, writing, computer, smartphones and iPads) (hours/day)	0.370^**^	<0.001
Time of watching TV (hours/day)	−0.048	0.608
Distance between eyes and TV	−0.133	0.155
The continuous-time of eye using	0.013	0.891
The rest time after continuous eye using	−0.254^**^	0.007
Ways of light exposure	0.235^*^	0.014
Outdoor light exposure (hours/day)	−0.242^*^	0.045
Indoor light exposure (through balcony overlooking)	−0.042	0.785
Total sleep time	−0.197^*^	0.040

* *Correlation is significant at the 0.05 level*,

** *correlation is significant at the 0.01 level*.

### Correlation Analysis of Asthenopia on Axial Elongation

**Figure 2:**
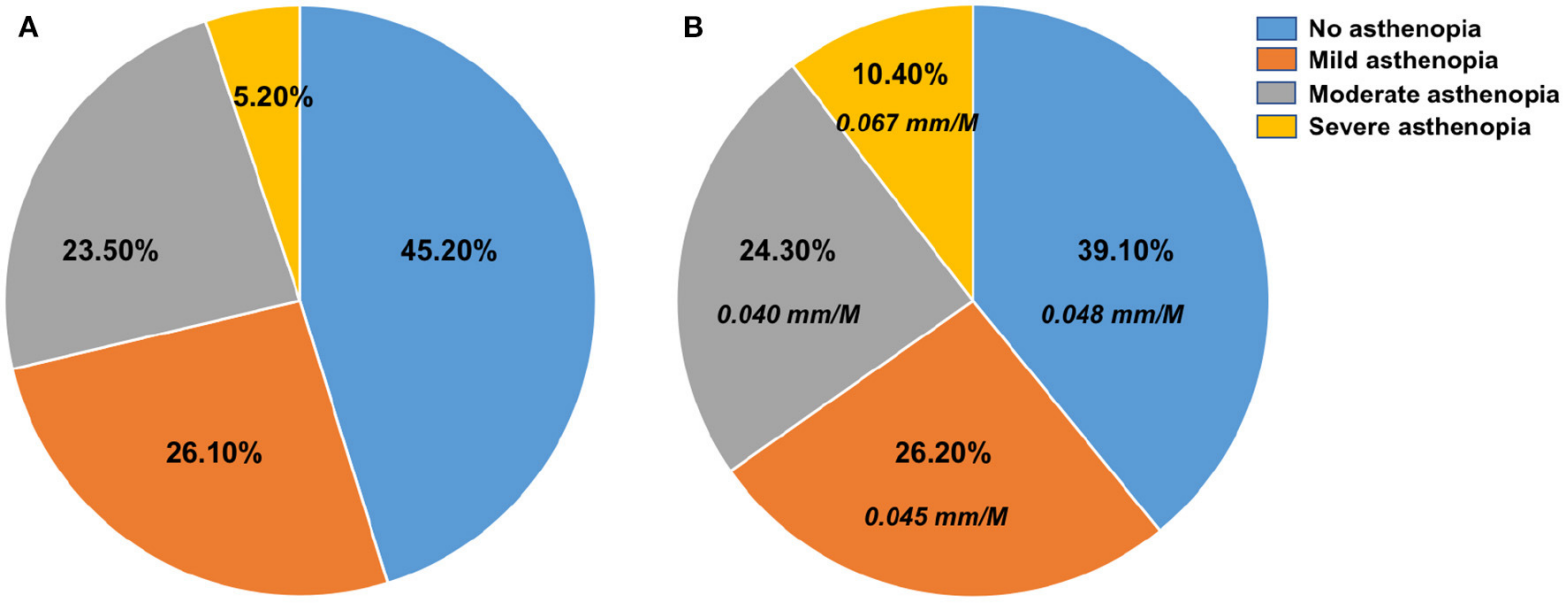
Proportion of asthenopia before the COVID-19 pandemic and after the strict home confinement during the COVID-19 pandemic **(A)** asthenopia before the COVID-19 pandemic; **(B)** asthenopia after the strict home confinement during COVID-19 pandemic and monthly growth rate of eye axial length in different degrees asthenopia.

Axial elongation positively correlated with severe asthenopia (*r* = 0.711, *P* = 0.01), but did not correlate with non-asthenopia, mild asthenopia, and moderate asthenopia. In addition, the axial growth rate of severe asthenopia was significantly faster than other degrees of asthenopia (*P* < 0.05; Figure 2B). The proportion of different degrees of asthenopia was significantly higher than that before the COVID-19 pandemic; particularly, the severe asthenopia doubled (*Z* = −2.322, *P* = 0.020; Figures 2A,B).

### Correlation Analysis of Visual Function on Axial Elongation

Visual function examination showed that the values of NRA, PRA, and AMP were lower than normal standard. The correlation analysis of visual function on myopia progression showed that the axial growth rate positively correlated with PRA (*r* = 0.333, *P* < 0.001) but negatively correlated with NRA (*r* = −0.127, *P* = 0.020) (Table 3). In addition, the visual function tests showed that 54 teenagers had normal AMP level and 61 teenagers' AMP levels were below normal, and the eye axial-length growth rate in children with lower AMP was 36.6% faster than children with normal AMP (0.056 vs. 0.041 mm/month, *P* = 0.01).

**Table 3:** Correlation analysis of visual function on the growth rate of eye axial length.

	**PRA**	**NRA**	**AMP**	**AC/A**	**Accommodation**
*r*	0.333^**^	−0.217^*^	−0.147	−0.044	−0.044
*P*	<0.001	0.020	0.117	0.640	0.640

* *Correlation is significant at the 0.05 level*,

** *correlation is significant at the 0.01 level*.

## Discussion

During the COVID-19 pandemic, the government-implemented measures of school closure and home confinement greatly affected the lifestyle and eye care habits of most children and adults. Abundant researches show that COVID-19 lockdown increases “quarantine dry eye.” Tear film integrity is known to be important in immune and visual processes, which can affect ocular surface health and visual quality (8, 9). Besides, most children use the smartphones, computers, or televisions to study and finish homework online via digital platforms. Compared to traditional school teaching methods, which allow children to alternate using their eyes at near and far distances, online studying switches learning to high-intensity, close-up use of the eyes. In addition, home confinement leads to less outdoor activity, causing children to spend more free time using digital products. These changes of lifestyle have greatly increased the exposure time to electronic devices, creating greater risks of myopia progression (10–13).

In this study, we discussed the progression of myopia during the strict home confinement measures implemented during the COVID-19 outbreak. The results showed that during the critical period when schools were generally closed (during the COVID-19 outbreak peak), the monthly axial elongation was 35% higher than that before COVID-19 (0.046 vs. 0.033 mm/month, *p* = 0.003), which was consistent with previous results (14–17). Chang et al. (14) found that the rate of spherical equivalent refractive (non-cycloplegic autorefraction) changes before and after the COVID-19 quarantine was −0.030 and −0.074 D/month. Xu et al. (15) found that the half-year incidence rate of myopia increased from 8.5% before the COVID-19 quarantine to 13.62% after the COVID-19 quarantine. To explore the factors influencing the increase incidence of myopia during the COVID-19 outbreaks, we used a questionnaire and a visual function examination to assess the effects of regional differences, inheritance factors, outdoor light exposure time, digital equipment exposure time and distance, types of digital devices, rest time and visual function on the development of myopia. As the results reveal, for the development of myopia, age and rest time after continuous eye-use, light exposure, sleep time and the distance from the screen when using a computer were protective factors. Notably, heredity, close indoor work and using of electronic products were risk factors. Like previous results, in addition to risk factors such as inheritance factors, too much close work and digital screen exposure time, and less light exposure time (3, 5, 14, 16, 18–22), new related factors such as the rest time after continuous eye-use and sleep duration were also exposed during the strict home confinement measures during the COVID-19 outbreak.

Digital platform learning during the COVID-19 pandemic has significantly increased exposure time to electronic screens, which is undoubtedly a risk factor for myopia. Previous studies on digital-screen use and myopic symptoms found that every 1-h increase in daily digital-screen use is associated with a 1.26 odds ratio higher risks of myopic progression, and that computers and smartphones are more likely to lead to myopia than TV (19), which is consistent with our findings. From the questionnaire, we found that watching TV was not correlated with the development of myopia (*r* = −0.133, *p* = 0.155); however, handheld digital devices, such as laptops, smartphones and iPads positively correlated with the development of myopia (*r* = 0.191, *p* = 0.041; *r* = 0.227, *p* = 0.021), which may be due to the closer distances of use of handheld digital devices compared to TV. Nowadays, it has been proved that the use of handheld digital devices can change the accommodation process (increased lag and concomitant reduced amplitude) and reduce fusional convergence, causing digital eye strain (23). In this study, CISS results showed that the severe asthenopia increased significantly, approximately doubling (10.4% vs. Baseline. 5.2%). Besides, severe asthenopia affects the progression of myopia and has a faster axial growth rate. We speculated that the increased screen exposure time and close-work time during COVID-19 are important factors in severe asthenopia. Besides, visual function results showed that the regulatory functions such as NRA, PRA, and AMP all decreased. Absolute values of these regulatory function indexes were negatively correlated with the axial length elongation, in other words, poor regulatory functions can promote the progression of myopia. Zhao et al. confirmed that the regulatory response induced by close work, which leads to a thinning of the choroid thickness and reduced choroid blood volume; this leads to hypoxia in adjacent scleral tissues, a decrease in extracellular matrix, and the accelerated the progression of myopia (24).

In addition, after the COVID-19 pandemic outbreak, people were quarantined at home to prevent further spread, reducing outdoor activities. While many parents made up for the lack of outdoor activities in innovative ways (such as terrace/ balcony views, and increased illumination of the learning environment), decreased outdoors activities remained an important risk factor for the development of myopia. About the questionnaire, we found that there was a positive correlation between the methods of light exposure and the development of myopia, with outdoor light exposure being a powerful protective measure for the development of myopia. Contrastingly, the balcony/terrace light exposure or the increased light intensity of the learning environment had no correlation with the development of myopia. This suggests that adequate outdoor activities are still vital to the prevention and control of myopia, whether during the COVID-19 pandemic outbreak or post-COVID-19 period. The increase of outdoor light increases the production and release of dopamine, which is beneficial to reduce axial elongation (25–28).

In conclusion, during home confinement measures implemented during the COVID-19 outbreak, the axial length monthly elongation was 35% higher than that before the COVID-19 pandemic. Furthermore, asthenopia increased significantly, severe asthenopia affected the progression of myopia, with a faster axial growth rate. In addition, the decreased outdoor activities and increased exposure time to electronic products, the less rest time after continuous eye-use and sleep time all played important roles in the development of myopia during the COVID-19 pandemic. Although this study had some limitations, in particular, there may be some memory bias when filling out the questionnaire. The results still have guiding significance for the prevention and control of myopia in the post-COVID-19 pandemic period. Notably, sufficient outdoor activity and less exposure time to digital screens are still important measures for the prevention and control of myopia.

## Data Availability Statement

The original contributions presented in the study are included in the article/Supplementary Material, further inquiries can be directed to the corresponding author/s.

## Ethics Statement

This study was approved by the Ethics Committee of Shandong Eye Institute. Written informed consent to participate in this study was provided by the participants' legal guardian/next of kin.

## Author Contributions

TC and LZ: wrote the manuscript and done the experiment. LK: consulted literature. XD: designed the experiments, reviewed, and edited the manuscript. All authors contributed to the article and approved the submitted version.

## Funding

This work was supported by the Qingdao Science and Technology Project (19-6-1-24-nsh).

## Conflict of Interest

The authors declare that the research was conducted in the absence of any commercial or financial relationships that could be construed as a potential conflict of interest.

## Publisher's Note

All claims expressed in this article are solely those of the authors and do not necessarily represent those of their affiliated organizations, or those of the publisher, the editors and the reviewers. Any product that may be evaluated in this article, or claim that may be made by its manufacturer, is not guaranteed or endorsed by the publisher.
