# Supplementary material for: Complex Interplay Between COVID-19 Lockdown and Myopic Progression
Source: Front Med (Lausanne). 2022 Mar 21;9:853293. doi: 10.3389/fmed.2022.853293 (PMC8978626; doi:10.3389/fmed.2022.853293)
Supplement: Supplementary file 1 [file Table_1.DOCX]

Table 1S. General characteristics of the subjects

| Parameter | Group | % |
| --- | --- | --- |
| Parental myopia history | Neither | 9.6 |
|  | One | 41.7 |
|  | Both | 48.7 |
| The myopia degree of parent (choose the higher) | <-3.00 D | 29.6 |
|  | -3.00 D to -6.00 D | 54.8 |
|  | -6.00 D to -9.00 D | 12.1 |
|  | >-9.00 D | 3.5 |
| Indoor reading and writing time (hours/day) | 0 to 2 h | 12.2 |
|  | 2 to 4 h | 47.0 |
|  | 4 to 6 h | 24.3 |
|  | >6 h | 16.5 |
| The time of using computer (hours/day) | 0 to 2 h | 50.5 |
|  | 2 to 4 h | 30.4 |
|  | 4 to 6 h | 17.4 |
|  | >6 h | 1.7 |
| The time of using smartphones and ipads (hours/day) | <1 h | 44.7 |
|  | 1 to 2 h | 33.0 |
|  | 2 to 4 h | 16.5 |
|  | 4 to 6 h | 3.9 |
|  | >6 h | 1.9 |
| The time of close work (including writing, computer, smartphones, ipads) (hours/day) | <2 h | 7.8 |
|  | 2 to 4 h | 29.6 |
|  | 4 to 6 h | 37.4 |
|  | >6 h | 25.2 |
| Rest time after continuous eye using | <10 min | 37.0 |
|  | 10 to 15 min | 42.3 |
|  | 15 to 20 min | 9.9 |
|  | 20 to 30 min | 5.4 |
|  | >30 min | 5.4 |
| The time of outdoor light exposure (hours/day) | <0.5 h | 10.1 |
|  | 0.5 to 1 h | 36.2 |
|  | 1 to 2 h | 40.6 |
|  | >2 h | 13.1 |
| Total sleep time | 6 to 8 h | 27.5 |
|  | >8 h | 72.5 |
